# Supplementary material for: Cross-feeding promotes heterogeneity within yeast cell populations
Source: Nat Commun. 2024 Jan 10;15:418. doi: 10.1038/s41467-023-44623-y (PMC10781747; doi:10.1038/s41467-023-44623-y)
Supplement: Supplementary file 1 — Supplementary Information [file 41467_2023_44623_MOESM1_ESM.pdf]

Cross-feeding promotes heterogeneity within yeast cell populations

Kevin K.Y. Hu[1], Ankita Suri[1], Geoff Dumsday[2], Victoria S. Haritos[1,*].

[1] Department of Chemical and Biological Engineering, Monash University, Clayton, VIC 3800, Australia

[2] Commonwealth Scientific and Industrial Research Organisation, Clayton, VIC 3169, Australia

*Corresponding author:

Assoc Prof Victoria S. Haritos,

Email: victoria.haritos@monash.edu

Phone: +61 3 9905 6873

Supplementary Information

Table S1. Plasmids used in this research project

Property: pESC-Selection marker X; Gene A<*<$Promoter_{\alpha}$|$Promoter_{\beta}$>*>Gene B

Plasmid	Properties	Source
***Saccharomyces cerevisiae* plasmids**		
pU3-GAL1_mT2	pESC-URA3; P_{GAL1}>*>*mTurquoise2*	This study
pU3-GAL1_mVenus	pESC-URA3; P_{GAL1}>*>*mVenus*	This study
pU3-GAL1_mKOκ	pESC-URA3; P_{GAL1}>*>*mKOκ*	This study
pU3-TEF1_mT2	pESC-URA3; P_{TEF1}>*>*mTurquoise2*	This study
pU3-CUP1_mT2	pESC-URA3; P_{CUP1}>*>*mTurquoise2*	This study
pU3d-GAL1_mT2	pESC-URA3d; P_{GAL1}>*>*mTurquoise2*	This study
pU3dCL1-GAL1_mT2	pESC-URA3dCL1; P_{GAL1}>*>*mTurquoise2*	This study
pL2-GAL10_mCh	pESC-LEU2; P_{GAL10}>*>*mCherry*	This study
pL2d-GAL10_mCh	pESC-LEU2d; P_{GAL10}>*>*mCherry*	This study
pKH+ve	pESC-URA3; *mTurquoise2*<*<P_{GAL1}|P_{TEF1}>*>*tetR*	This study
pKH-ve	pESC-LEU2d; $P_{GAL1[tetOx3]}$>*>*mCherry*	This study
*represents a Kozak sequence AAACA that was added at the 5' of the start codon of all fluorescent reporter genes (i.e. *mTurquoise2*, *mVenus*, *mKOκ* and *mCherry*) as well as the *tetR* gene to enhance their recombinant expression in *S. cerevisiae* [1].		

Table S2. *Saccharomyces cerevisiae* and *Escherichia coli* strains used in this research project. Genotype: Notable chromosomal modifications stated either in full form following the mating type (e.g. MATa *his3Δ1 leu2Δ0 met15Δ0 ura3Δ0*) or by strain name (e.g. BY4741) or in a mixed form (e.g. BY4741*fur4Δ*); plasmid(s) transformed into the chassis (if applicable).

Strain	Genotype	Source
***Saccharomyces cerevisiae* strains**		
BY4741	MATa *his3Δ1 leu2Δ0 met15Δ0 ura3Δ0*	Gifted by Prof. Hongyuan Yang, UNSW
BY4741*urk1Δ*	BY4741 *YNR012W::kanMX4*	[2,3]
BY4741*fur4Δ*	BY4741 *YBR021W::kanMX4*	[2,3]
BY4741*fui1Δ*	BY4741 *YBL042C::kanMX4*	[2,3]
BY_mTU3	BY4741; pU3-GAL1_mT2	This study
BY_mVeU3	BY4741; pU3-GAL1_mVenus	This study
BY_mKOU3	BY4741; pU3-GAL1_mKOκ	This study
BY_TEF1mTU3	BY4741; pU3-TEF1_mT2	This study
BY_CUP1mTU3	BY4741; pU3-CUP1_mT2	This study
BY_mTU3d	BY4741; pU3d-GAL1_mT2	This study
BY_mTU3dCL1	BY4741; pU3dCL1-GAL1_mT2	This study
BY*fur4Δ*_mTU3	BY4741*fur4Δ*; pU3-GAL1_mT2	This study
BY*fur4Δ*_mTU3d	BY4741*fur4Δ*; pU3d-GAL1_mT2	This study
BY*fur4Δ*_mTU3dCL1	BY4741*fur4Δ*; pU3dCL1-GAL1_mT2	This study
BY*fui1Δ*_mTU3	BY4741*fui1Δ*; pU3-GAL1_mT2	This study
BY4741*fur4Δfui1Δ*	BY4741*fur4Δ fui1::HIS3*	This study
BYdbKO_mTU3d	BY4741*fur4Δfui1Δ*; pU3d-GAL1_mT2	This study
BYdbKO_mTU3dCL1	BY4741*fur4Δfui1Δ*; pU3dCL1-GAL1_mT2	This study
BY_mChL2	BY4741; pL2-GAL10_mCh	This study
BY_mChL2d	BY4741; pL2d-GAL10_mCh	This study
BY_KH	BY4741; pKH+ve; pKH-ve	This study

yAG1	MATa *BUD4-S288C can1-100 leu2Δ::*P_{ACT1}*-ymCitrine-*T_{ADH1}*-His3MX6 ura3Δ0*	4
yAG2	MATa *ade1-14 ADE2 bar1 BUD4-S288C can1-100 cyh2r his3-11 leu2Δ::HphMX4::ymCherry trp1-1 ura3-1*	4
yAG19	MATa *cin2Δ::LEU2 his3Δ200 leu2-3,112* P_{ACT1}*::ymCherry-kanMX4 ura3-52*	4
yAG20	MATa *can1-100 cdc26Δ::URA3 his3-11,15 leu2-3,112* P_{ACT1}*:ymCitrine-kanMX4 trp1-1 ura3-1*	4
***Escherichia coli* strains**		
BW25113	*rrnB3 ΔlacZ4787 hsdR514 Δ(araBAD)567 Δ(rhaBAD)568 rph-1*	5
BW25113*pyrFΔ*	BW25113 *pyrF::kanR*	5

<u>Table S3</u>. Modification made on the basis of the commercial pESC vector series

Promoter sequence		
**Promoter**	**Sequence (5'→3')**	**Source**
P_{TEF1}	ccacacaccatagcttcaaaatgtttctactcctttttactcttccagattttctc ggactccgcgcatcgccgtaccacttcaaaacacccaagcacagcatact aaatttcccctctttcttcctctagggtgtcgttaattacccgtactaaaggtttgg aaaagaaaaaagagaccgcctcgtttctttttcttcgtcgaaaaaggcaata aaaatttttatcacgtttctttttcttgaaaattttttttttgatttttttctctttcgatga cctcccattgatatttaagttaataaacggtcttcaatttctcaagtttcagtttca tttttcttgttctattacaactttttttacttcttgctcattagaaagaaagcatagca atctaatctaag	*Saccharomyces* Genome Database (SGD)
P_{CUP1}	ctagttagaaaaagacatttttgctgtcagtcactgtcaagagattcttttgctg gcatttcttctagaagcaaaaagagcgatgcgtcttttccgctgaaccgttcc agcaaaaaagactaccaacgcaatatggattgtcagaatcatataaaag agaagcaaataactccttgtcttgtatcaattgcattataatatcttcttgttagtg caatatcatatagaagtcatc	*Saccharomyces* Genome Database (SGD)
P_{URA3}	ttcaattcatcattttttttttattcttttttttgatttcggtttctttgaaatttttttgattcg gtaatctccgaacagaaggaagaacgaaggaaggagcacagacttag attggtatatatacgcatatgtagtgttgaagaaacatgaaattgcccagtatt cttaacccaactgcacagaacaaaaacctgcaggaaacgaagataaat c	pESC-URA3 Vector (Agilent Technologies, Catalog # 217454)
P_{URA3d}	ggaaacgaagataaatc	[6]
P_{LEU2}	aactgtgggaatactcaggtatcgtaagatgcaagagttcgaatctcttagc aaccattatttttttcctcaacataacgagaacacacaggggcgctatcgca cagaatcaaattcgatgactggaaattttttgttaatttcagaggtcgcctgac gcatatacctttttcaactgaaaaattgggagaaaaaggaaaggtgagag cgccggaaccggcttttcatatagaatagagaagcgttcatgactaaatgct tgcatcacaatacttgaagttgacaatattatttaaggacctattgtttttccaat aggtggttagcaatcgtcttactttctaacttttcttaccttttacatttcagcaata tatatatatatatttcaaggatataccattcta	pESC-LEU2 Vector (Agilent Technologies, Catalog # 217452)
P_{LEU2d}	ccaataggtggttagcaatcgtcttactttctaacttttcttaccttttacatttcag caatatatatatatatatttcaaggatataccattcta	[7]
$P_{GAL1[tetOx3]}$	atattgaagtatccctatcagtgatagagaagtccctatcagtgatagagaa tccctatcagtgatagagatgcgtcctcgtcttcaccggtcgcgttcctgaaa cgcagatgtgcctcgcgccgcactgctccgaacaataaagattctacaata ctagcttttatggttatgaagaggaaaaattggcagtaacctggccccacaa accttcaaatgaacgaatcaaattaacaaccataggatgataatgcgatta gtttttttagccttatttctggggtaattaatcagcgaagcgatgatttttgatctatt aacagatatataaatgcaaaaactgcataaccactttaactaatactttcaa cattttcggtttgtattacttcttattcaaatgtaataaaagtatcaacaaaaaat tgttaatatacctctatactttaacgtcaaggagaaaaaac	Modified from $P_{[tetO]3\text{-in-GAL1}}$ created by Hsu and colleagues [8]
C-terminal destabilizing sequence		
**Degron**	**Sequence (N-ter → C-ter)**	**Source**
CL1	ACKNWFSSLSHFVIHL	[9]

Selection marker and their modified derivatives				
**Selection marker**	**Properties**		**Selection marker**	**Properties**
URA3	P_{URA3}>*URA3*		LEU2	P_{LEU2}>*LEU2*
URA3d	P_{URA3d}>*URA3*		LEU2d	P_{LEU2d}>*LEU2*
URA3dCL1	P_{URA3d}>*URA3*>CL1			

<u>Table S4</u>. List of Primers and PCR fragments used in this research project. Sequences complimentary to the DNA template are in *ITALIC*.

#	**PCR fragment**	**Primer name**	**Template**	**Sequence (5' → 3')**
1	*fui1::HIS3* disruption PCR fragment	FUI1HIS_KO_FW	pIYC04 [10]	*ACTTTGCAGCACATTGCGGAAATAAAAGGCGGTAACTAGTCCTCTCATTC*aacacggcattagtcaggga
		FUI1HIS_KO_RV		*GGCTTTGACATTATCATGATAAGGTGCTACTTGCGTTGTTAAC*ttcacaccgcatagatccgt
2	*fui1::HIS3* check PCR fragment	FUI1HIS_KO_Check_FW	*FUI1* from BY4741 genomic DNA	*GGCGTTCACTGTGGTTGATG*
		FUI1HIS_KO_Check_RV		*CTGAAGACCTATGGTAAACAAGAC*

<u>Table S5</u>. Instrument settings and sorted sample details of the FACS experiment

**Instrument details**				
Data Source: Cytometer<br>Nozzle Diameter (µm): 70<br>Sheath Pressure (PSI): 60<br>Sort Device: 2 Tube Holder - 2 Way Sort<br>Piezo Amplitude: 5.20<br>Drop Delay: 49.1000		Sort Mode: 1.0 Drop Pure<br>Drop Envelope: 1.0 Drop<br>Sort Objective: Purify<br>Phase Mask: 16/16<br>Extra Coincidence Bits: 4<br>Drop Frequency (kHz): 98.60		
**Sorted sample details** (mTur2 – [405] 450/50; PI – [488] 670/30)				
**Name**	**Sort Count**	**Sort Rate**	**Abort Rate**	**Efficiency**
mTur2-ve	1.32e7	1.48e3	151	90.7%
mTur2+ve	2.64e7	2.96e3	249	92.2%
FACS Ctrl	2.70e7	9.98e3	532	94.9%

Table S6. **Calibration curves and other quantitative data four pyrimidine nucleosides and bases**

Analyte	Calibration curve	Range (mg L^{-1})	Retention time (min)	Correlation coefficient
Uracil	Abs = 174.18X - 6.0523	0.1 - 100	13.4	1
Uridine	Abs = 90.136x + 2.9868	0.1 - 100	18.9	1
Cytosine	Abs = 115.87x - 2.1917	0.1 - 100	9.4	1
Cytidine	Abs = 71.61x + 1.4488	0.1 - 100	15.6	1

Table S7. **Relative fluorescence output of BY_mTU3, BY_mTU3d and BY_mTU3dCL1 cell cultures after being cultivated for 24 h in 2% galactose + 1% raffinose SC-Ura media (n = 3)**

Strain	TECAN measurement (0.2 mL of cells with $O.D._{600\ nm}$ of 0.4)			Normalised Fluorescence per $O.D._{600\ nm}$
	$Abs_{600\ nm}$ (a.u.)	$Em_{475\ nm}$ (a.u.)	$Em_{475\ nm}$ / $Abs_{600\ nm}$	
BY_mTU3	0.165 ± 0.006	872 ± 23.2	5280	1.00
BY_mTU3d	0.161 ± 0.012	1768 ± 104.1	11010	2.09
BY_mTU3dCL1	0.161 ± 0.015	3031 ± 121.6	18797	3.56

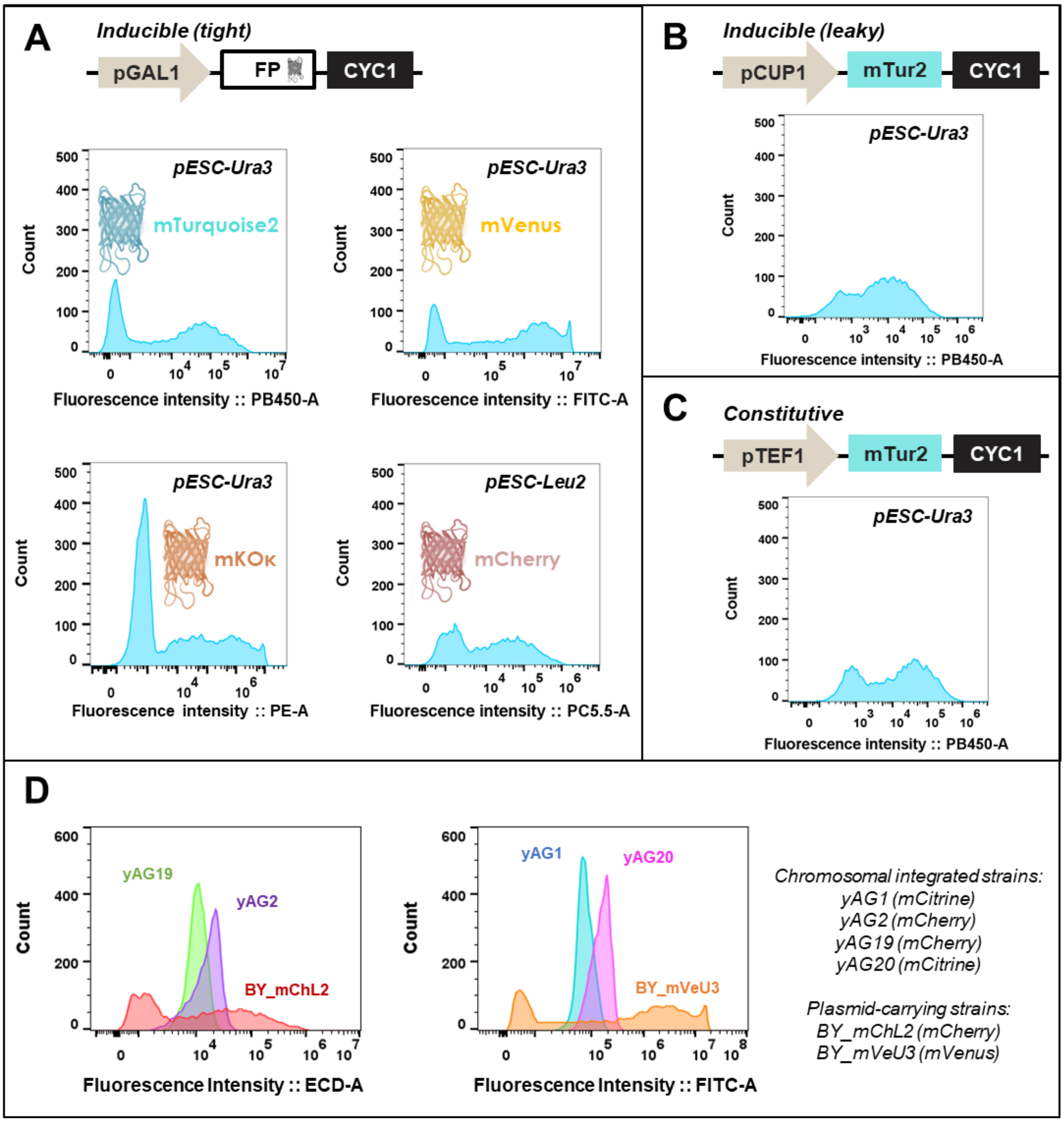

Figure S1. Flow cytometry fluorescence expression profiles of various yeast strains that either carry the fluorescent reporter gene on plasmids (A,B and C) or have it chromosomally integrated (D). Panel A, B and C display the fluorescence profiles of yeast strains with reporter gene harbored on plasmids and under the regulation of either pGAL1 [A: BY_mTU3 (mTurquoise2), BY_mVeU3 (mVenus), BY_mKOU3 (mKOκ) and BY_mChL2 (mCherry)], pCUP1 [B: BY_CUP1mTU3 (mTurquoise2)] or pTEF1 [C: BY_TEF1mTU3 (mTurquoise2)]. (D) Left panel: Expression profiles of yAG2 and yAG19, both have mCherry chromosomally integrated, in comparison to BY_mChL2 which has mCherry harboured on pESC-LEU2 plasmid; Right panel: Expression profiles of yAG1 and yAG20, both have mCitrine chromosomally integrated, in comparison to BY_mVeU3 which has mVenus harboured on pESC-URA3 plasmid. All plasmid-carrying cells were cultivated in 2% galactose + 1% raffinose SC-X selective media, wherein X is either Ura or Leu, as appropriate. All chromosomal integrated (yAG) strains were cultivated in 2% glucose YPD non-selective media. All samples were collected at late exponential - early stationary phase. Single experiments conducted for each.

A

B

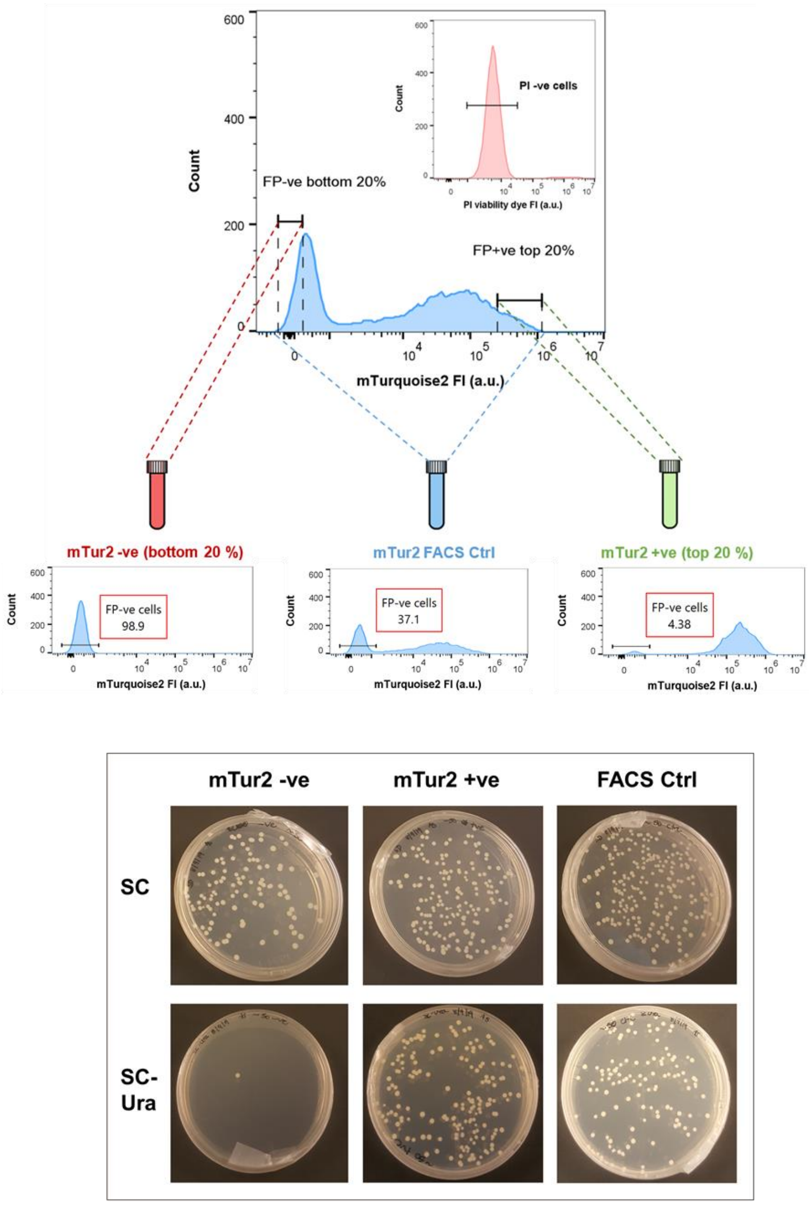

Colony count		mTur2 -ve	mTur2 +ve	FACS Ctrl
	2% Glu SC plate	107	182	264
	2% Glu SC-Ura plate	1	172	126

Figure S2. Culturability of the FP-ve (mTur2-ve) and FP+ve (mTur2+ve) subpopulations of an exponentially growing BY_mTU3 cell culture after being separated through fluorescence activated cell sorting (FACS) and re-cultivated on SC and SC-Ura agar plates supplemented with 2% glucose. A sample ungated against FP intensity (FACS Ctrl) was included as a comparator. The FACSed mTur2-ve sample contained 98.9% FP-ve cells; the FACSed mTur2+ve sample contained 4.38% FP-ve cells; The FACS Ctrl sample contained 37.1% FP-ve cells. All cells within the FACSed samples were PI-ve. The BY_mTU3 cell culture was cultivated in 2% galactose + 1% raffinose SC-Ura selective media for 16 h prior to the FACS process. Cells were sorted back into 2% galactose + 1% raffinose SC-Ura selective media prior to plating on the SC and SC-Ura agar plates. Based on their O.D.$_{600\ nm}$ readings, the FACSed samples were diluted with PBS and aliquoted onto each agar plate to a nominal CFU count within the range of 50-500 per plate, and for each FACSed sample the volume and concentration of cells being plated on the SC-Ura and SC plates were kept the same. Single experiment conducted.

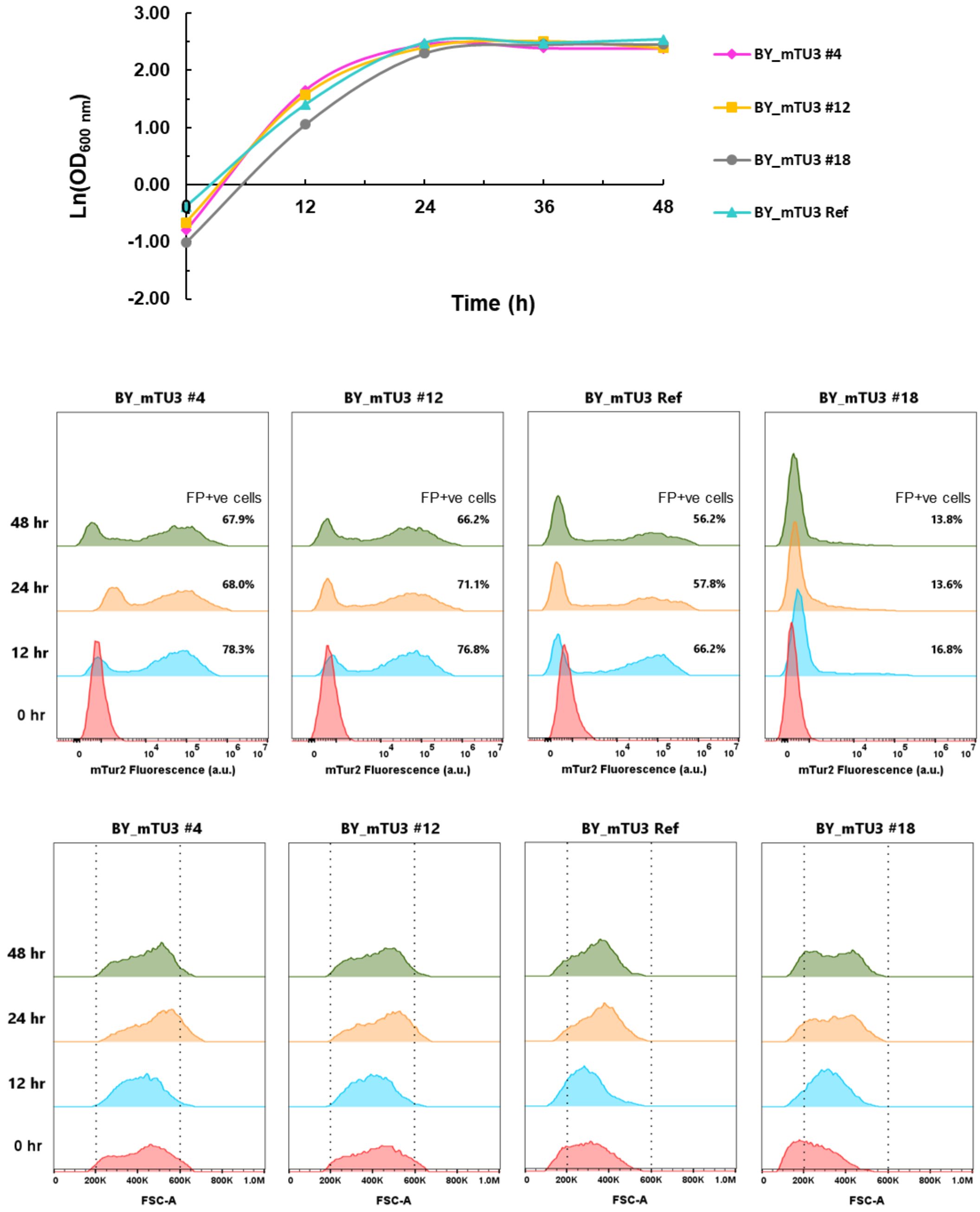

Figure S3. Comparison of (A) growth, (B) population mTur2 expression profiles and (C) cell size distribution profiles between that of a normal BY_mTU3 clone (BY_mTU3 Ref) versus the mutant clones (#4, #12 and #18) when cultivated in 2% galactose + 1% raffinose SC-Ura liquid media over 48 h shake flask batch mode cultivation (n = 1). Despite being able to grow comparatively well in SC-Ura selective media, #18 failed to express much recombinant protein (B), while the proportion of FP+ve cells in #4 and #12 are greater than that of the non-mutated BY_mTU3 Ref population at all sampled time points (B). In addition, median cell size of #4 and #12 mutants were also larger than BY_mTU3 Ref population (C).

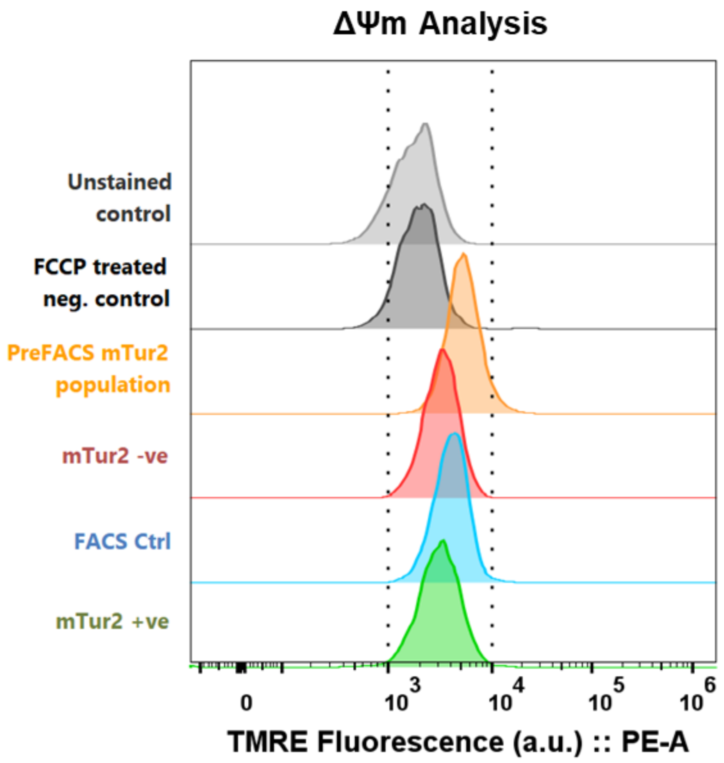

Figure S4. Comparison in terms of mitochondrial membrane potential ΔΨm between the mTur+ve, mTur-ve and FACS Ctrl samples (n=1) immediately after the FACS process as measured using flow cytometry (PE-A channel, 585/42 nm). The BY_mTU3 cell culture was cultivated in SC-Ura selective media containing 2% galactose + 1% raffinose for 16 h prior to cell sorting**.** Cells were sorted back into fresh SC-Ura selective media containing 2% galactose + 1% raffinose and subsequently stained with TMRE (tetramethylrhodamine, ethyl ester) dye following the supplier's protocols prior to flow cytometry analysis.

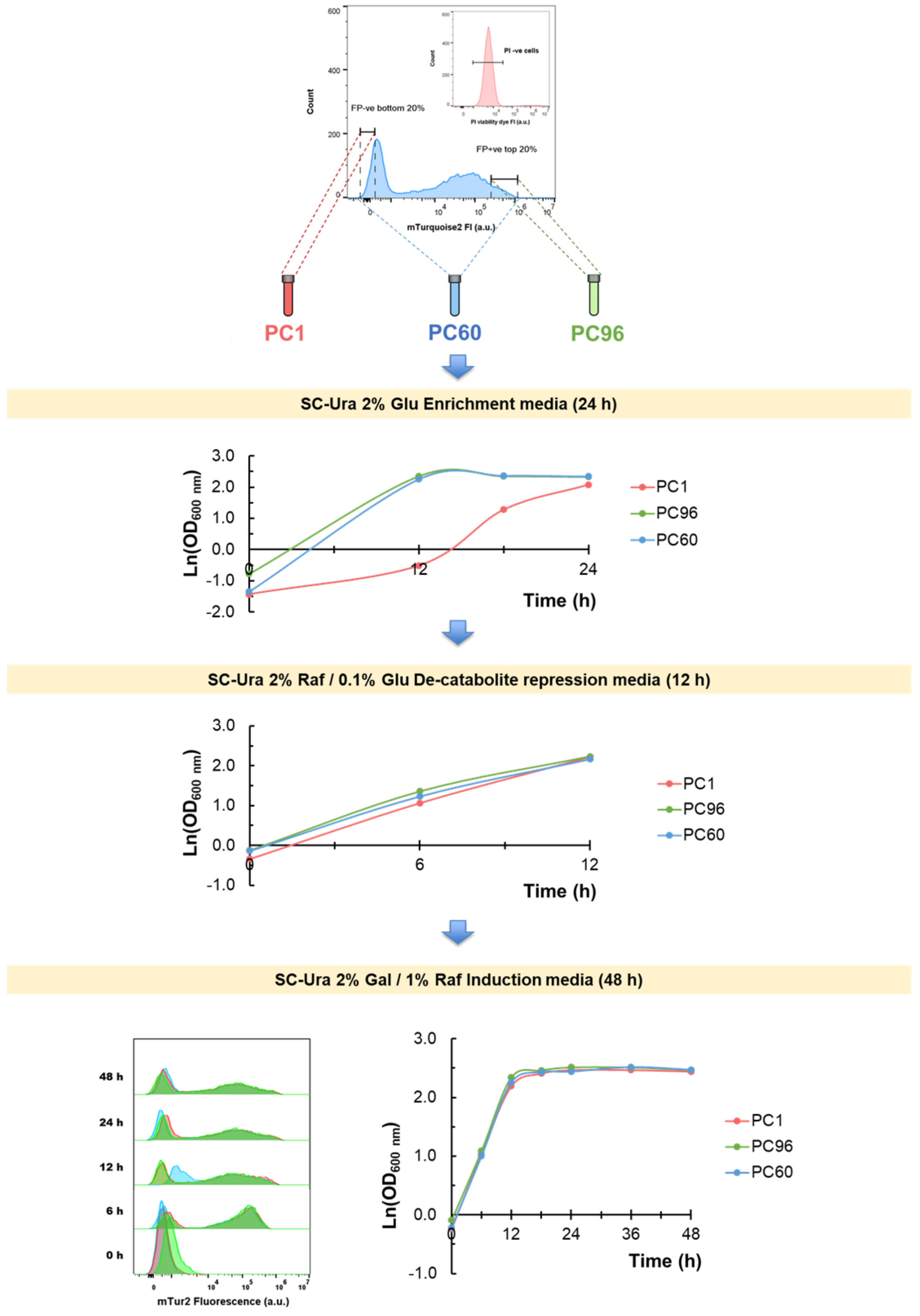

Figure S5. Growth curves of three BY_mTU3 populations containing different initial fraction of plasmid-carrying cells (PC1 = 1%, PC60 = 60% and PC96 = 96%, respectively), when being cultivated in SC-Ura media under batch mode (n = 1). The FACSed populations were initially propagated in SC-Ura + 2% Glu media for 24 h and subsequently in SC-Ura + 2% Raf + 0.1% Glu media for another 12 h prior to galactose induction. The mTur2 expression profiles of the three populations at the galactose-induction stage were also monitored (samples were displayed with the same colour-code as in growth curves). The initial BY_mTU3 cell culture was cultivated in 2% galactose + 1% raffinose SC-Ura selective media for 16 h prior to cell sorting.

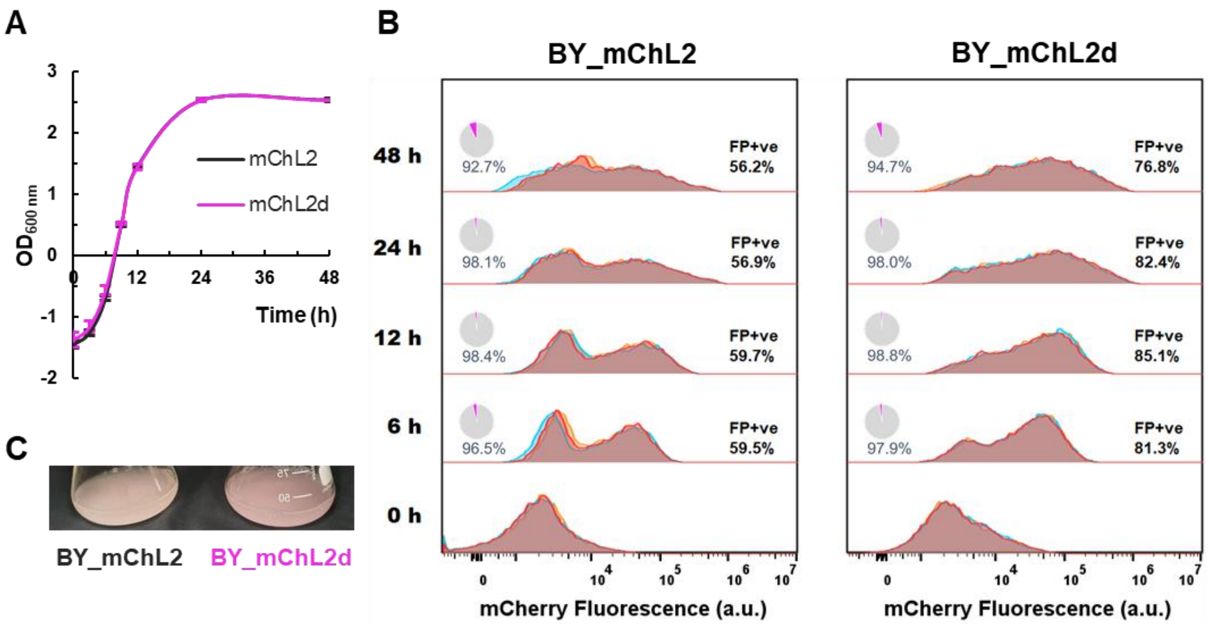

Figure S6. (A) Growth curves (data points were presented as mean values ± s.d.), (B) mCherry expression profiles and (C) visual appearance of the BY_mChL2 and BY_mChL2d cell cultures when being cultivated in SC-Leu media supplemented with 2% galactose + 1% raffinose under batch mode (n = 3 independent cell cultures propagated from different clones). Pie chart insets in B represent the percentage of viable, PI-negative cells (grey portion) within the culture at each sampled time point.

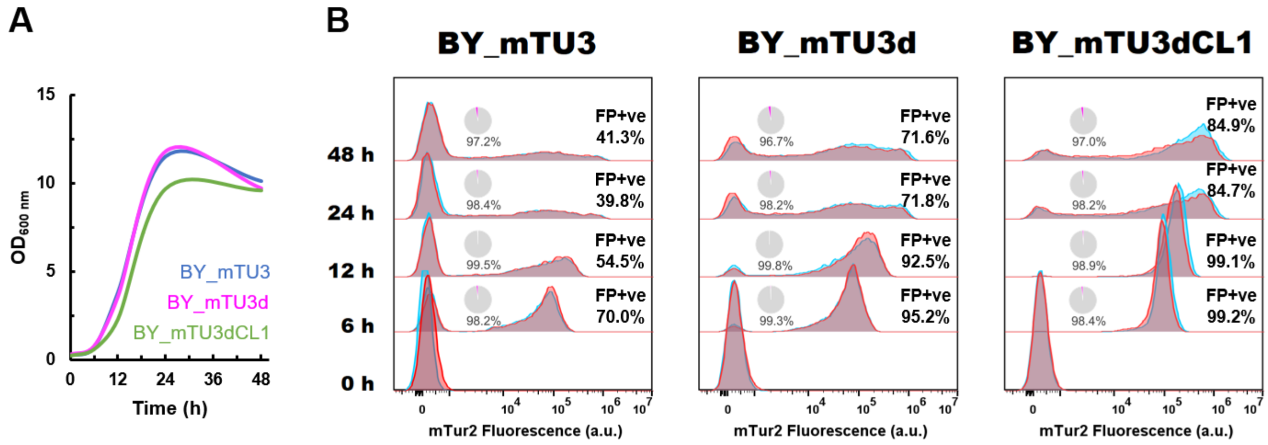

Figure S7. (A) Growth curves and (B) mTur2 expression profiles of BY_mTU3, BY_mTU3d and BY_mTU3dCL1 populations when being cultivated in SC non-selective media supplemented with 2% galactose + 1% raffinose under batch mode (n = 2 independent cell cultures propagated from different clones). Pie chart insets in B represent the percentage of viable, PI-negative cells (grey portion) within the culture at each sampled time point.

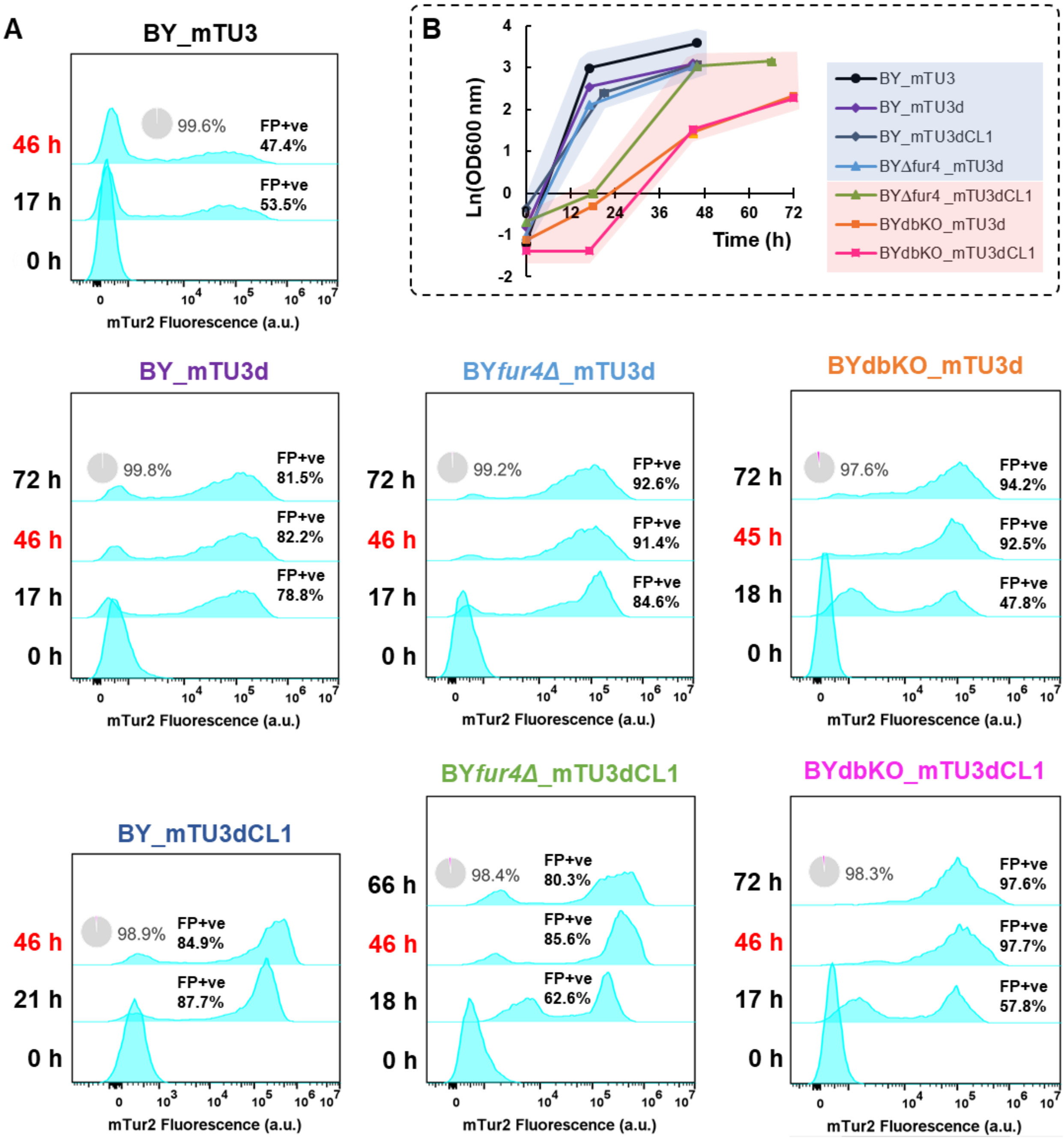

C

Strain	FP+ve subpopulation @ 45 - 46 h			
	%	mTur2 recombinant expression		Cell size (Median FSC-A)
		MFI (a.u.)	rCV	
BY_mTU3	47.4	3.51×10^4	192	4.34×10^5
BY_mTU3d	82.2	8.24×10^4	131	4.04×10^5
BY_mTU3dCL1	84.9	2.52×10^5	97.1	4.57×10^5
BY*fur4Δ*_mTU3d	91.4	7.60×10^4	118	3.21×10^5
BY*fur4Δ*_mTU3dCL1	85.6	1.92×10^5	83.1	3.27×10^5
BYdbKO_mTU3d	92.5	6.58×10^4	110	2.39×10^5
BYdbKO_mTU3dCL1	97.7	1.01×10^5	117	2.60×10^5

Figure S8. (A) mTur2 expression profiles and (B) growth curves of various recombinant yeast strains when cultivated in non-selective (YPD) media (n = 1). Pie chart insets in A represent the percentage of viable, PI-negative cells (grey portion) within the culture at selected time point. All yeast strains were seeded in corresponding selective media [SC-Ura for BY4741 and BY4741*fur4Δ* strains; SC-Ura-His for BY4741*fur4Δfui1Δ* (BYdbKO) strains] supplemented with 2% raffinose + 0.1% glucose prior to being induced in YPD media containing 2% galactose. Growth fitness of the yeast strains can be categorised into two groups: one with an observable initial long lag phase (shaded in red) and one without (shaded in blue). (C) Single-cell measurement statistics regarding mTur2 expression and cell size of the examined yeast strains at 45 – 46 h post-induction in YPD media.

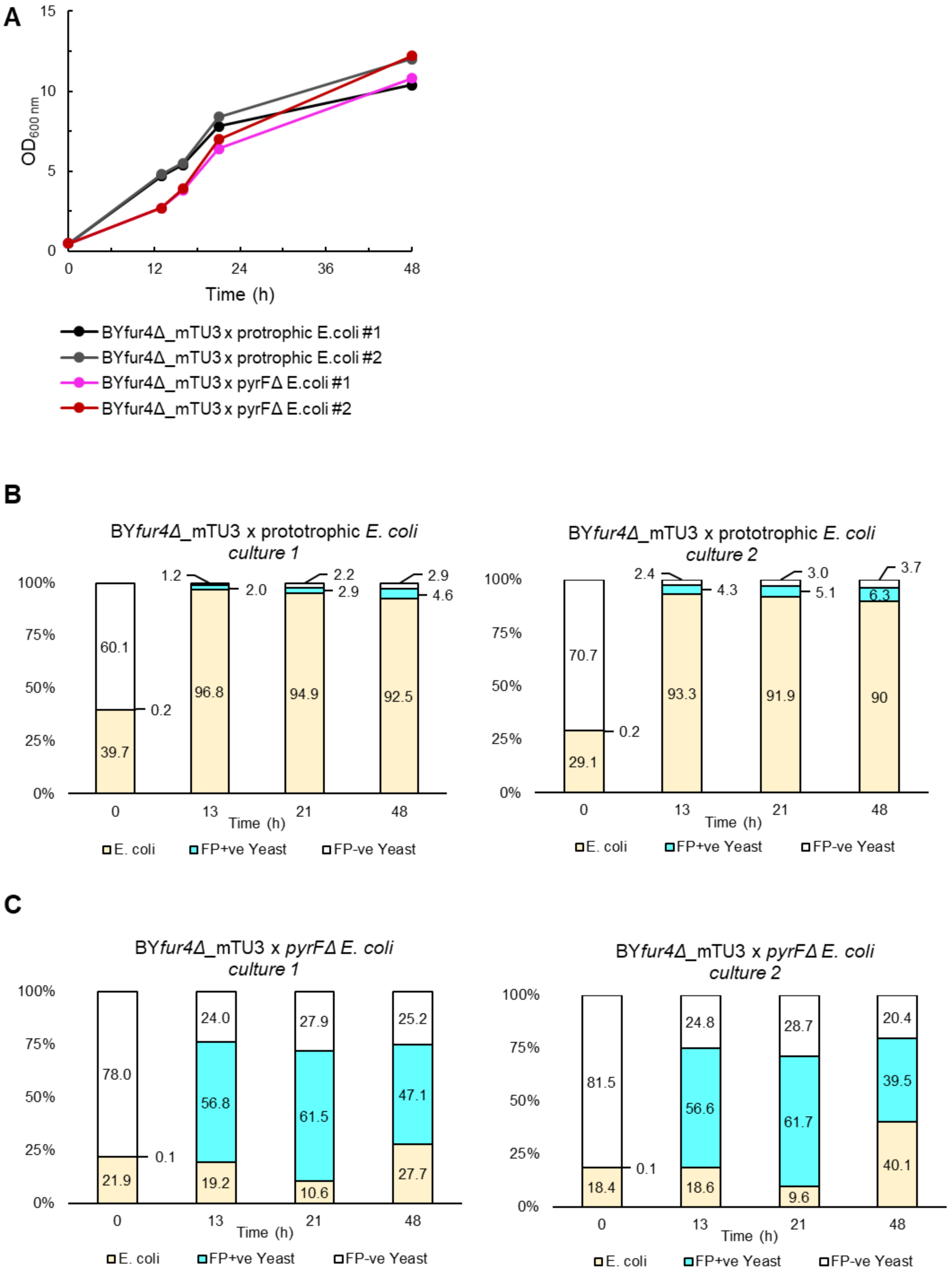

Figure S9. (A) Growth curves of co-cultures of the yeast BY4741*fur4Δ*_mTU3 with either prototrophic or pyrimidine auxotrophic (*pyrFΔ*) *E. coli* in modified SC-Ura media supplemented with 2% galactose and 1% raffinose over 48 h of batch mode fermentation. Compositions of the co-cultures between BY4741*fur4Δ*_mTU3 and prototrophic *E. coli* are presented in B, while the compositions of co-cultures between BY4741*fur4Δ*_mTU3 and *pyrFΔ E. coli* are presented in C.

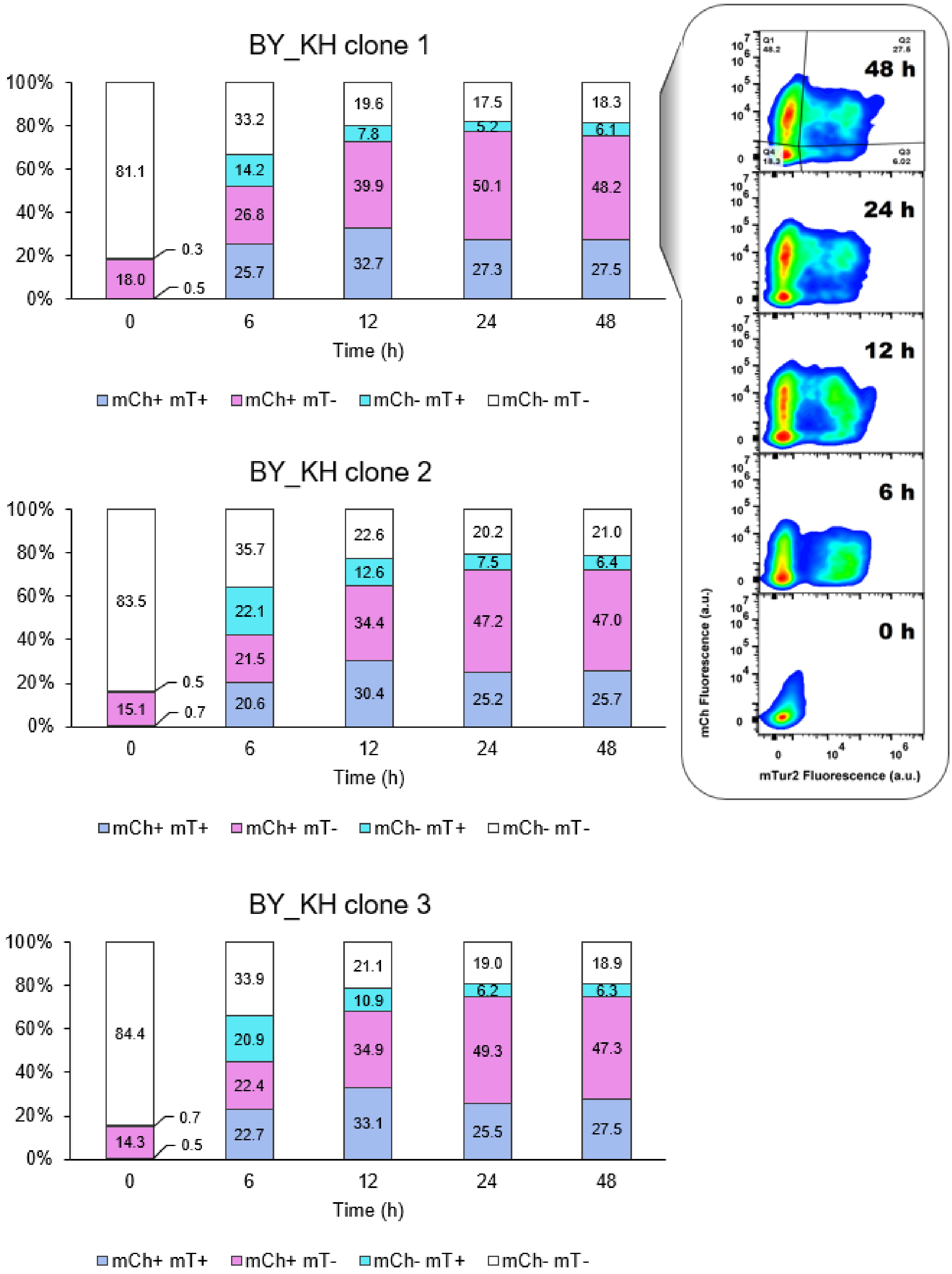

Figure S10. Subpopulation compositions derived from three different BY_KH clones when cultivated in SC-Ura-Leu media supplemented with 2% galactose and 1% raffinose over 48 h of batch mode fermentation. Insets are mTurquoise2 vs mCherry fluorescent intensities (a.u.) bivariate plots of BY_KH culture 1 across the sampled timepoints.

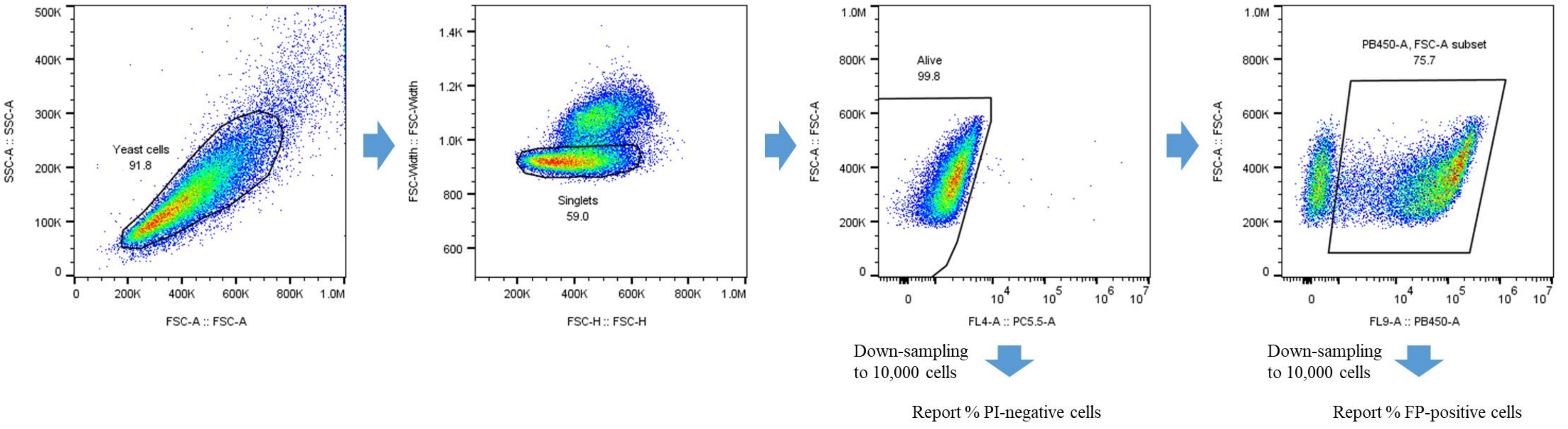

<u>Figure S11</u>. Gating strategies applied for yeast flow cytometry data processing. mTurquoise2 was shown as an example reporting fluorophore. To minimise cross-sample variation in the report of fluorescence intensity, only singlet / non-budding *S. cerevisiae* cells were included in the reported flow cytometry data of yeast monocultures. Additionally, unless otherwise noted, only cells with intact cell membrane and inferentially healthy were included in the reported flow cytometry data, this is because cells with permeable membranes were by default FP-ve due to recombinant protein leakage [11]. The FI threshold which separates the FP-ve and FP+ve subpopulations is always mapped to the relative position of the FP-ve subpopulation instead of adhering to a fixed FI threshold. This is because the auto-fluorescence of yeast cells, which constitutes the “fluorescence” of the FP-ve cells, would change in accordance to the physiological conditions of the cells, which in turns would be influenced by environmental factors that are known to change along a batch fermentation process.

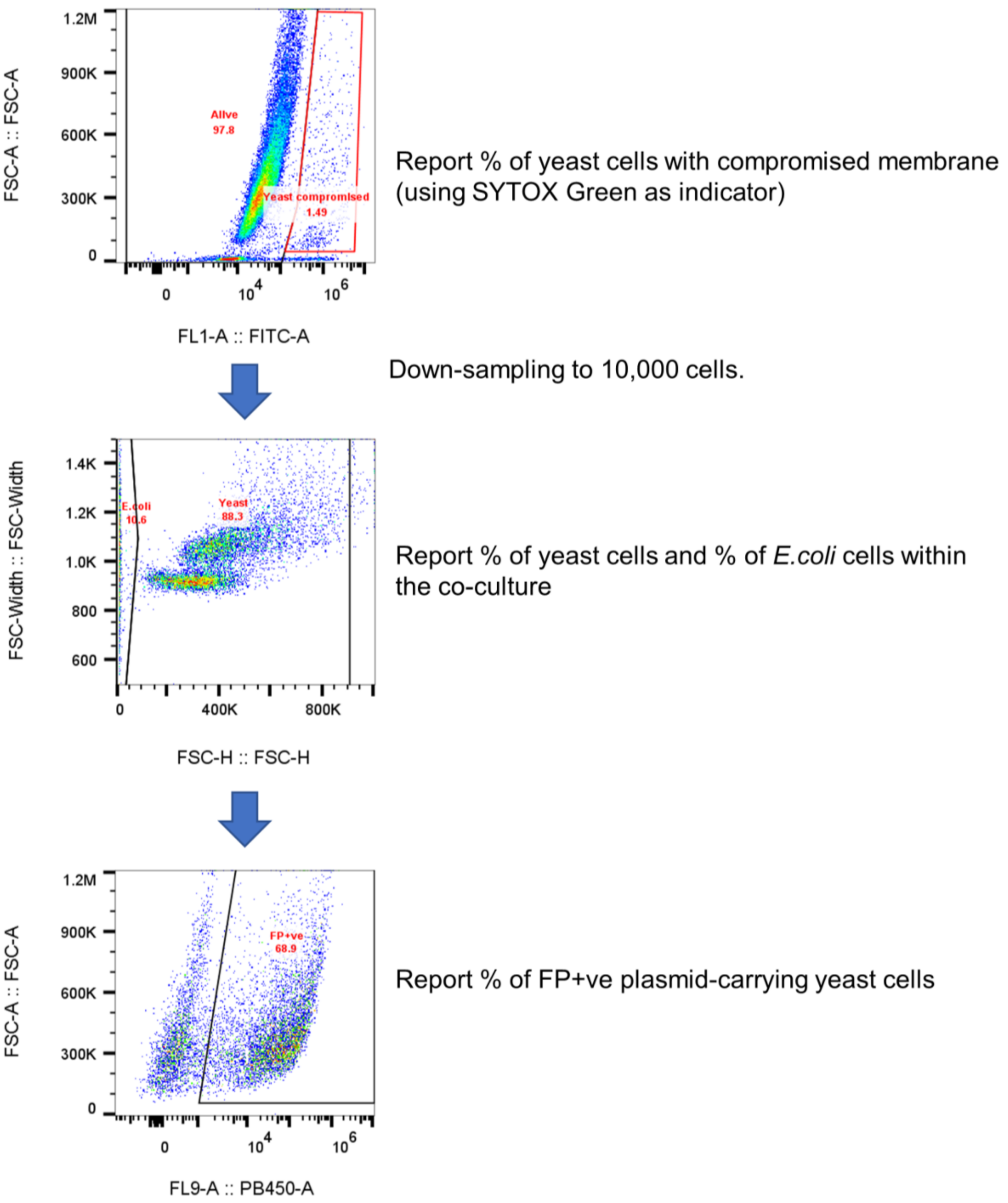

Figure S12. Gating strategies applied for *S. cerevisiae* – *E. coli* co-culture flow cytometry data processing. Only cells with intact cell membrane and inferentially healthy were included in the reported flow cytometry data. Cells with permeable membranes were by default mTur2-negative due to recombinant protein leakage [11]. To reduce the complexity associated with cell counting, doubling cells were not stratified from singlets in flow cytometry data of samples obtained from yeast-bacteria co-cultures, that is all doublets are counted as “1” instead of “2” cells.

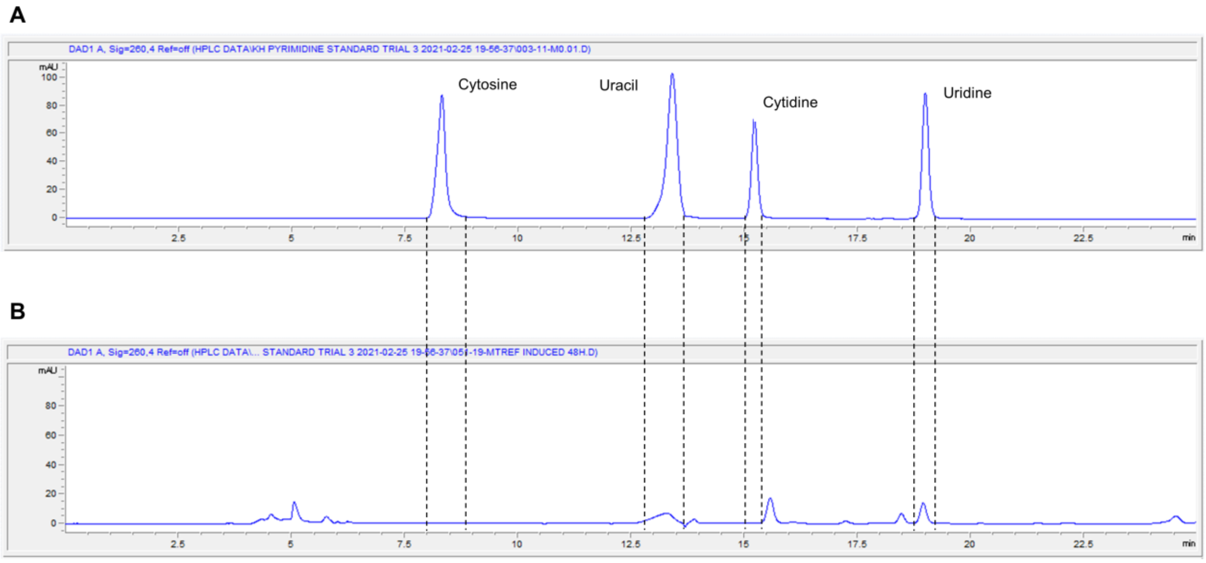

Figure S13. HPLC chromatograms of A) a standard mixture of cytosine, cytidine, uracil and uridine each with the concentration of 0.01 g L^{-1}; and B) a fermentation broth sample taken from a BY_mTU3 population after being cultivated for 48 h in 2% galactose + 1% raffinose SC-Ura media. Retention time of analytes: Cytosine = 9.4 min, Uracil = 13.4 min, Cytidine = 15.6 min, Uridine = 18.9 min.

Supplementary References

1. Runguphan, W. & Keasling, J. D. Metabolic engineering of Saccharomyces cerevisiae for production of fatty acid-derived biofuels and chemicals. *Metab. Eng.* **21**, 103–113 (2014).

2. Giaever, G. *et al.* Functional profiling of the Saccharomyces cerevisiae genome. *Nature* **418**, 387–391 (2002).

3. Winzeler, E. A. *et al.* Functional characterization of the S. cerevisiae genome by gene deletion and parallel analysis. *Science (80-.).* **285**, 901–906 (1999).

4. Giometto, A., Nelson, D. R. & Murray, A. W. Physical interactions reduce the power of natural selection in growing yeast colonies. *Proc. Natl. Acad. Sci. U. S. A.* **115**, 11448–11453 (2018).

5. Baba, T. *et al.* Construction of Escherichia coli K-12 in-frame, single-gene knockout mutants: the Keio collection. *Mol. Syst. Biol.* **2**, 2006.0008 (2006).

6. Okkels, J. S. A URA3-Promoter Deletion in a pYES Vector Increases the Expression Level of a Fungal Lipase in Saccharomyces cerevisiae. *Ann. N. Y. Acad. Sci.* **782**, 202–207 (1996).

7. Seresht, A. K., Nørgaard, P., Palmqvist, E. A., Andersen, A. S. & Olsson, L. Modulating heterologous protein production in yeast: The applicability of truncated auxotrophic markers. *Appl. Microbiol. Biotechnol.* **97**, 3939–3948 (2013).

8. Hsu, C. *et al.* Stochastic signalling rewires the interaction map of a multiple feedback network during yeast evolution. *Nat. Commun.* **3**, (2012).

9. Gilon, T., Chomsky, O. & Kulka, R. G. Degradation signals for ubiquitin system proteolysis in Saccharomyces cerevisiae. *EMBO J.* **17**, 2759–2766 (1998).

10. Chen, Y., Daviet, L., Schalk, M., Siewers, V. & Nielsen, J. Establishing a platform cell factory through engineering of yeast acetyl-CoA metabolism. *Metab. Eng.* **15**, 48–54 (2013).

11. Delvigne, F. *et al.* Green fluorescent protein (GFP) leakage from microbial biosensors provides useful information for the evaluation of the scale-down effect. *Biotechnol. J.* **6**, 968–978 (2011).
