# Supplementary material for: Cross-feeding promotes heterogeneity within yeast cell populations
Source: Nat Commun. 2024 Jan 10;15:418. doi: 10.1038/s41467-023-44623-y (PMC10781747; doi:10.1038/s41467-023-44623-y)
Supplement: Supplementary file 3 — Reporting Summary [file 41467_2023_44623_MOESM3_ESM.pdf]

nature portfolio

Corresponding author(s): Victoria Haritos
Last updated by author(s): 17-12-2023

Reporting Summary

Nature Portfolio wishes to improve the reproducibility of the work that we publish. This form provides structure for consistency and transparency in reporting. For further information on Nature Portfolio policies, see our Editorial Policies and the Editorial Policy Checklist.

Statistics

For all statistical analyses, confirm that the following items are present in the figure legend, table legend, main text, or Methods section.

n/a	Confirmed	
[ ]	[x]	The exact sample size (*n*) for each experimental group/condition, given as a discrete number and unit of measurement
[ ]	[x]	A statement on whether measurements were taken from distinct samples or whether the same sample was measured repeatedly
[ ]	[x]	The statistical test(s) used AND whether they are one- or two-sided<br>*Only common tests should be described solely by name; describe more complex techniques in the Methods section.*
[x]	[ ]	A description of all covariates tested
[x]	[ ]	A description of any assumptions or corrections, such as tests of normality and adjustment for multiple comparisons
[ ]	[x]	A full description of the statistical parameters including central tendency (e.g. means) or other basic estimates (e.g. regression coefficient) AND variation (e.g. standard deviation) or associated estimates of uncertainty (e.g. confidence intervals)
[ ]	[x]	For null hypothesis testing, the test statistic (e.g. *F*, *t*, *r*) with confidence intervals, effect sizes, degrees of freedom and *P* value noted<br>*Give P values as exact values whenever suitable.*
[x]	[ ]	For Bayesian analysis, information on the choice of priors and Markov chain Monte Carlo settings
[x]	[ ]	For hierarchical and complex designs, identification of the appropriate level for tests and full reporting of outcomes
[x]	[ ]	Estimates of effect sizes (e.g. Cohen's *d*, Pearson's *r*), indicating how they were calculated

Our web collection on statistics for biologists contains articles on many of the points above.

Software and code

Policy information about availability of computer code

Data collection	Flow cytometry data acquisition - CytExpert software v2.3 for CytoFlex V5-B3-R3 and CytExpert software v2.5 for CytoFlex V5-B3-Y5-R3<br>Fluorescence-activated cell sorting instrument control - BD FACS Sorftware v1.2.0.142<br>TECAN microplate reader data acquisition - i-control v1.11<br>HPLC instrument control and data acquisition - ChemStation LC 1220 + RID data system
Data analysis	Flow cytometry data analysis - FlowJo v10.6.1 – v10.8.0<br>HPLC data analysis - ChemStation LC (offline)<br>Other data analyses - Microsoft Excel (Office 16)

For manuscripts utilizing custom algorithms or software that are central to the research but not yet described in published literature, software must be made available to editors and reviewers. We strongly encourage code deposition in a community repository (e.g. GitHub). See the Nature Portfolio guidelines for submitting code & software for further information.

Data

Policy information about availability of data

All manuscripts must include a data availability statement. This statement should provide the following information, where applicable:

- Accession codes, unique identifiers, or web links for publicly available datasets
- A description of any restrictions on data availability
- For clinical datasets or third party data, please ensure that the statement adheres to our policy

The extended data figures and supplementary information supporting the findings of this work are enclosed along with this manuscript.
Source data are provided as a Source Data file. Raw and processed flow cytometry data are available upon request to the authors as this requires having the appropriate software to view and analyze. The data files are extremely large and can cause system crashes.

Research involving human participants, their data, or biological material

Policy information about studies with human participants or human data. See also policy information about sex, gender (identity/presentation), and sexual orientation and race, ethnicity and racism.

Reporting on sex and gender	n/a
Reporting on race, ethnicity, or other socially relevant groupings	n/a
Population characteristics	n/a
Recruitment	n/a
Ethics oversight	n/a

Note that full information on the approval of the study protocol must also be provided in the manuscript.

Field-specific reporting

Please select the one below that is the best fit for your research. If you are not sure, read the appropriate sections before making your selection.

[x] Life sciences [ ] Behavioural & social sciences [ ] Ecological, evolutionary & environmental sciences

For a reference copy of the document with all sections, see nature.com/documents/nr-reporting-summary-flat.pdf

Life sciences study design

All studies must disclose on these points even when the disclosure is negative.

Sample size	For all yeast and bacterial samples analysed via flow cytometry, minimum of 20,000-40,000 microorganisms, as a research standard protocol, such that the data could all be down-sampled to 10,000 microorganisms for each experimental test.
Data exclusions	No data was excluded from the presented results. For all flow cytometry data presented the gating strategies applied were provided in the supplementary information section.
Replication	All experiments were replicated with a minimum of biological triplicates except for data presented in Figure 4 d which were biological duplicates. In Figure 2 a, we present the result of physical cell sorting and subculturing of a standard experiment which had been reproduced in biological triplicates and the phenomena investigated in 65 different clones previously (Figure 1).
Randomization	In our experiments the microorganisms are engineered to express or have deleted genes for specific traits whose phenomena we were testing. Hence, the microorganisms could not be allocated to experiments randomly.
Blinding	For the reasons given above and because one trait under investigation, production of blue fluorescent protein, was easily observed by eye the samples were not blinded.

Reporting for specific materials, systems and methods

We require information from authors about some types of materials, experimental systems and methods used in many studies. Here, indicate whether each material, system or method listed is relevant to your study. If you are not sure if a list item applies to your research, read the appropriate section before selecting a response.

Materials & experimental systems

n/a	Involved in the study
☒	☐ Antibodies
☒	☐ Eukaryotic cell lines
☒	☐ Palaeontology and archaeology
☒	☐ Animals and other organisms
☒	☐ Clinical data
☒	☐ Dual use research of concern
☒	☐ Plants

Methods

n/a	Involved in the study
☒	☐ ChIP-seq
☐	☒ Flow cytometry
☒	☐ MRI-based neuroimaging

Flow Cytometry

Plots

Confirm that:

☒ The axis labels state the marker and fluorochrome used (e.g. CD4-FITC).

☒ The axis scales are clearly visible. Include numbers along axes only for bottom left plot of group (a 'group' is an analysis of identical markers).

☒ All plots are contour plots with outliers or pseudocolor plots.

☒ A numerical value for number of cells or percentage (with statistics) is provided.

Methodology

Sample preparation	Cells were resuspended or diluted with PBS to OD600nm of ~0.1-0.2 prior to analysis. Either propidium iodine (final conc. 5 µg/mL) or SYTOX Green (final conc. 1 µM), as appropriate, was added into the sample to select for healthy cells (with intact membrane). Samples subjected for mitochondrial membrane potential assay were prepared according to supplier's instructions.
Instrument	Flow cytometry: CytoFLEX V5-B3-R3 or CytoFLEX LX V5-B3-Y5-R3 (Beckman Coulter)<br>FACS: BD Influx System (BD Biosciences)
Software	Flow cytometry - CytExpert software v2.3 and v2.5<br>FACS - BD FACS Sortware v1.2.0.142<br>Data analysis - FlowJo v10.6.1 – v10.8.0
Cell population abundance	At least 10 millions cells were acquired for each sorted population. Post-sorting quality control was performed on flow cytometry and reported as the fraction of "negative" cells with the "positive" sorted subpopulation, and vice versa.
Gating strategy	Yeast mono-cultures - gate 1: FCS-A vs SSC-A was used to gate yeast cells over debris and contaminants, gate 2: FSC-W vs FSC-H was used to differentiate singlets over doublets and budding yeast cells, gate 3: FSC-A vs PC5.5-A (for PI) or FSC-A vs FITC-A (for SYTOX Green) was used to isolate singlets with intact cell membrane, which can then be further analysed regarding their phenotypes (e.g. fluorescent protein-producers or non-producers).<br><br>Yeast-bacteria co-cultures - gate 1: FSC-A vs FITC-A was used to gate out cells with compromised membrane, gate 2: FSC-W vs FSC-H to differentiate yeast cells over bacterial cells. The yeast cells can then be further analysed regarding their phenotypes (e.g. fluorescent protein-producers or non-producers).

☒ Tick this box to confirm that a figure exemplifying the gating strategy is provided in the Supplementary Information.
